# Multistate resistive switching in silver nanoparticle films

**DOI:** 10.1088/1468-6996/16/4/045004

**Published:** 2015-08-03

**Authors:** Eric J Sandouk, James K Gimzewski, Adam Z Stieg

**Affiliations:** 1 Department of Physics and Astronomy, UCLA, USA; 2 Department of Chemistry and Biochemistry, UCLA, USA; 3 California NanoSystems Institute (CNSI), UCLA, USA; 4 WPI Center for Materials Nanoarchitectonics (MANA), National Institute for Materials Science (NIMS), Japan; 5 School of Physics, Centre for Nanoscience and Quantum Information, University of Bristol, UK

**Keywords:** resistive switching, nanomaterials, memory

## Abstract

Resistive switching devices have garnered significant consideration for their potential use in nanoelectronics and non-volatile memory applications. Here we investigate the nonlinear current–voltage behavior and resistive switching properties of composite nanoparticle films comprising a large collective of metal–insulator–metal junctions. Silver nanoparticles prepared via the polyol process and coated with an insulating polymer layer of tetraethylene glycol were deposited onto silicon oxide substrates. Activation required a forming step achieved through application of a bias voltage. Once activated, the nanoparticle films exhibited controllable resistive switching between multiple discrete low resistance states that depended on operational parameters including the applied bias voltage, temperature and sweep frequency. The films’ resistance switching behavior is shown here to be the result of nanofilament formation due to formative electromigration effects. Because of their tunable and distinct resistance states, scalability and ease of fabrication, nanoparticle films have a potential place in memory technology as resistive random access memory cells.

## 1. Introduction

Nanoparticle based devices and related amorphous materials have been considered for their potential use in electronics as resistive random access memory (RRAM) or thin film transistors [1] because of versatility, low-temperature fabrication and multiple resistance states which are required for memory technology [2]. Such qualities may allow circumvention of forthcoming obstacles such as write/erase time, endurance, retention time and power consumption [2, 3] that modern memory technology would inevitably meet as it advances. Recently, there has been an increasing number of reports on resistive switching and memristive behavior observed in nanoparticle assemblies [4] and various proposed mechanisms for such behavior [5] in both uniform [6] and non-uniform [7] thin films. Because the precise operational mechanism is unknown, several models exist for electrical conductivity in films of nanoparticles linked by organic molecules [8], as well as cluster assembled [9] and stacked metal–metal oxide–metal devices [6, 9]. Metal–insulator–metal (MIM) based systems rely on metal ions dissolving and passing through the insulating layer to be reduced at the opposite electrode, forming a conductive bridge. Each junction forms a resistance switching random access memory cell, or RRAM cell [10].

In nanoparticle assemblies, observations of non-ohmic conductance have been attributed to either polymer breakdown between junctions [11] or filament formation between individual metal particles likely due to charge carrier migration across undoped regions with a moving boundary [4] or attractive van der Waals forces [5]. In many cases the nanoparticles are coated with an organic molecule or polymer through which resistive switching occurs via redox activity, the formation of a charge-transfer complex, or the formation of donor–acceptor couples [10], but the exact mechanism is often difficult to determine.

**Figure 1.**
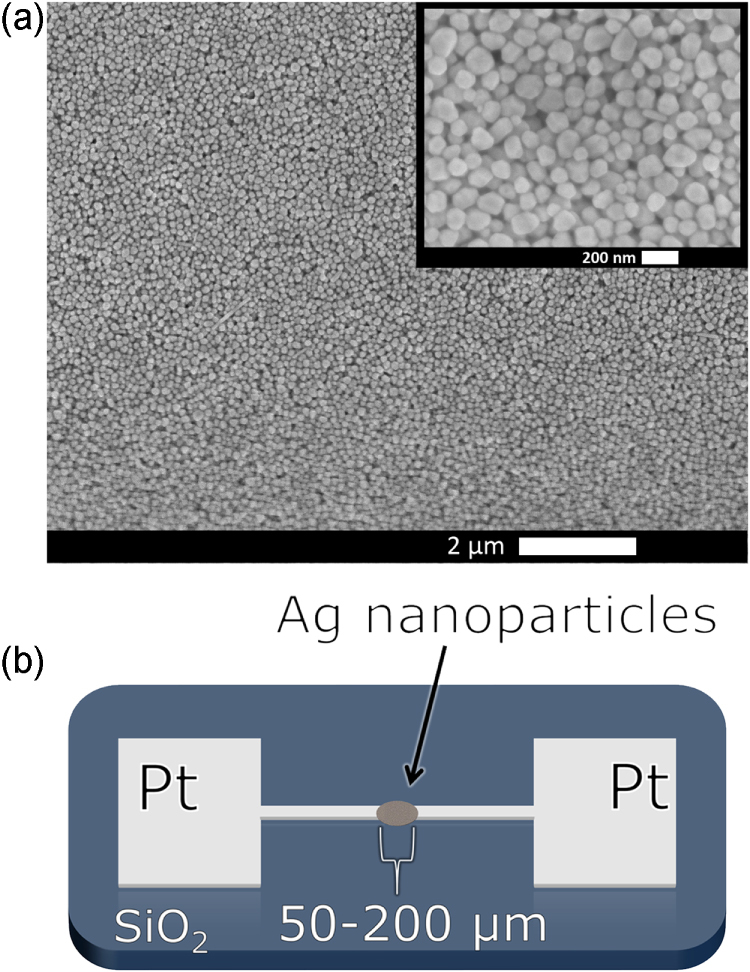
(a) Scanning electron microscopy image of a silver nanoparticles after synthesis, scale bar = 2 *μ*m. A higher magnification image is inset, scale bar = 200 nm. (b) Schematic of a nanoparticle film device (not drawn to scale). Platinum electrodes with terminals spaced 50–200 *μ*m apart on a SiO_2_ substrate surround the drop-caste nanoparticles on either side.

The breakage of nanoscale metallic structures during the application of external bias is often caused by electromigration [5, 12–14] which, in nanoparticle assemblies, creates sub-nanometer gaps between the metallic bridges that link neighboring particles [14]. The effects of electromigration are manifested as voids which can cause open circuit failures or hillocks which can extrude material from adjacent conducting surfaces, even breaking through protective coating layers and causing short-circuit failures. It is understood that spatial variations in atomic density, current density, average grain size, etc promote divergence in atomic or vacancy flux [15]. In the films produced via the polyol process, there is considerable spatial variation due to the non-uniformity in shape and size of silver nanoparticles (figure 1(a)).

Techniques for the fabrication of nanoparticle films and devices typically involve chemical solution deposition followed by UV irradiation treatment [6] or require advanced instrumentation such as ultra-high vacuum atomic cluster deposition [16]. Here we demonstrate an accessible approach capable of producing nanoparticle films that exhibit nonlinear current–voltage behavior and the cause of this behavior being electromigration-induced metallic filament formation is investigated. The requirements [2] for memory applications that cells contain at least two metastable resistance states that can be switched by external stimuli and clearly distinguished during read-out are satisfied by the resistive switching systems presented here. The films possess at least two stable ON-states that can be controlled by varying the frequency of applied bias or by changing the operating temperature in addition to an OFF-state, for a total of at least three distinguishable resistance states.

## 2. Experimental

Silver nanoparticles were synthesized by dissolving of silver nitrate (Alfa Aesar, 99.9+%) in ethylene glycol (EG, Alfa Aesar, 99%) at a weight/volume ratio of 1:4 and added to an equal volume of a preheated polyvinyl-pyrrolidone (PVP) [17] (TCI, 40 000 g mol^−1^) and EG mixture with a weight/volume ratio of 1:2 and continuously purged with nitrogen gas (99.5% purity) to avoid unwanted effects from reactions with the air. The solution was then heated and maintained at 150–160 °C under stirring at 100 rpm for 4 h. An initially pale yellow EG–PVP–AgNO_3_ mixture soon turned dark brown, indicating the presence of silver nanoparticles. After cooling to room temperature, the particles were removed from solution by centrifugation, rinsed with ethanol to remove excess PVP and suspended in methanol. Tetraethylene glycol (TTEG) was added to the concentrated nanoparticle suspension (1:10 *μ*L), allowing particles to remain spatially separated following solvent evaporation.

The nanoparticle suspension was then drop-cast onto a silicon substrate (525 *μ*m thickness; p-type; 100 mm diameter; 500 nm thermal oxide) with pre-patterned Cr/Pt (15/150 nm) electrodes and dried in air. Electrical characterization of the films employed an NI USB-6259 BNC module (National Instruments) for current–voltage (*I*–*V*) spectroscopy. Structural characterization was performed using optical and scanning electron (FEI Nova600 NanoLab) microscopes. Data analysis and visualization were executed using MATLAB R2010b (MathWorks) and OriginPro 9.1 (OriginLab Corporation), respectively.

## 3. Results and discussion

**Figure 2.**
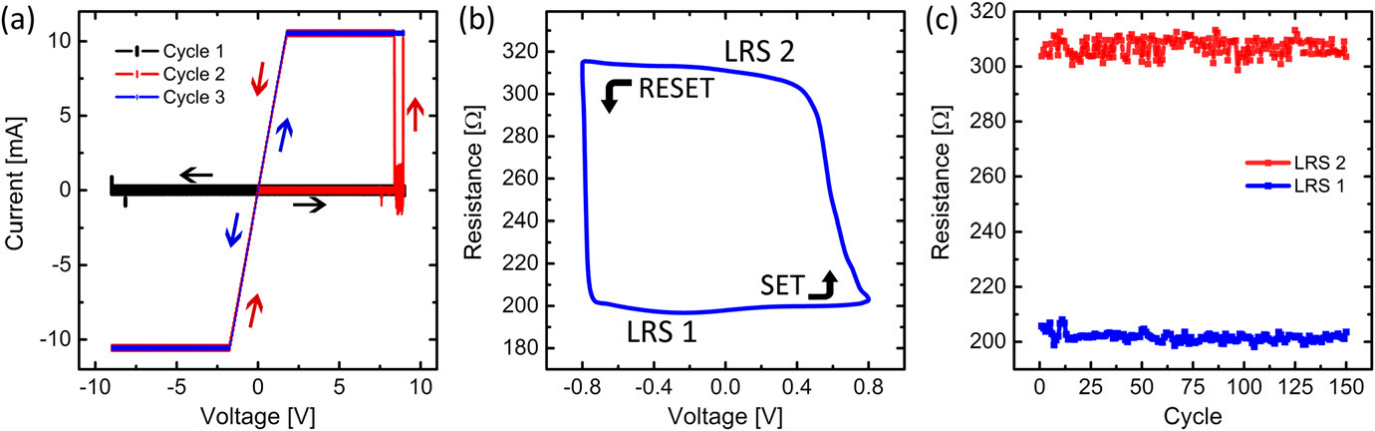
(a) The activation process was performed by applying repeated cycles of ±9 V, 5 Hz sine wave bias (sampling frequency = 10 kHz) at room temperature. (b) After activation, the devices exhibited hysteretic *I–V* behavior with two distinct states: LRS-1 with a resistance near 200 Ω and LRS-2 at roughly 310 Ω. (c) Stability of the two distinct LRS over repeated voltage cycling after the device was fully operational.

Like other filament-based resistive switching systems such as metal-oxide thin films [18] a forming step was required to activate the nanoparticle assemblies. The forming step creating the initial filamentary substructures that are responsible for conduction was executed by applying 7–9 V, 5 Hz sine wave voltage sweeps across the film (figure 1(b)). During the initial OFF/ON state transition (figure 2(a)), the resistance dropped by five to six orders of magnitude from an initial resistance of 10^8^ Ω. Though other filament and MIM-based systems such as atomic switch networks [19] or other nanowire networks [11] display a gradual increase in conductance during the forming sequence [10], these nanoparticle films exhibited an abrupt change in conductance within a single time step for our measurement system (100 *μ*s). A higher sampling rate, however, would have been able to precisely determine the switching time. Despite this more abrupt transition, the required forming step indicates that conduction in our nanoparticle assemblies is due to filament based processes.

Filament formation can result from strong electric fields being applied across these nanoparticle films. Large potential gradients across insulated gaps cause particle deformation in the direction of the applied field such that a percolating configuration is attained [20]. Considering the relative size of our nanoparticles (80–100 nm), which makes tunneling or van der Waals attraction [5] unlikely, we postulate that the combined effects of filament formation and breakage via electromigration are responsible for the spontaneous increase and decrease in conductance observed in our nanoparticle films.

Increased voltage inevitably causes a greater current density and concomitant Joule heating which in turn results in more severe electromigration effects. During the initial forming step for our nanoparticle assemblies, hillocks may be created with such geometries that the local electric fields responsible for conduction between neighboring particles are more effective at inducing mass flux divergence in the same direction [20] as initial hillock formation. Since local electric field strength is greater near sharp edges of a conducting surface, there is directional preference for electromigration effects—away from the vertices of the silver nanoparticle structures—which results in a preferential bias polarity that causes filament formation while the opposite polarity promotes breakage. Our experimental data (figure 2(b)) indicates the occurrence of this phenomenon as one polarity was associated with an increase in conductance which the opposite polarity caused a significant decrease.

The nanoparticle films exhibited highly nonlinear *I–V* behavior with resistance switching capabilities (figure 2). Monitoring the current output revealed the existence of at least two distinct low resistance states (LRS)—there was typically a stable state of 200–220 Ω termed LRS-1 and a second state, LRS-2, with higher resistance of 300–330 Ω (figure 2(b)). Switching between the two LRS was robust under repeated cycles of applied bias as shown in figure 2(c). During the experiment, no indication of any deviation from the presented behavior was observed after thousands of voltage cycles were applied.

**Figure 3.**
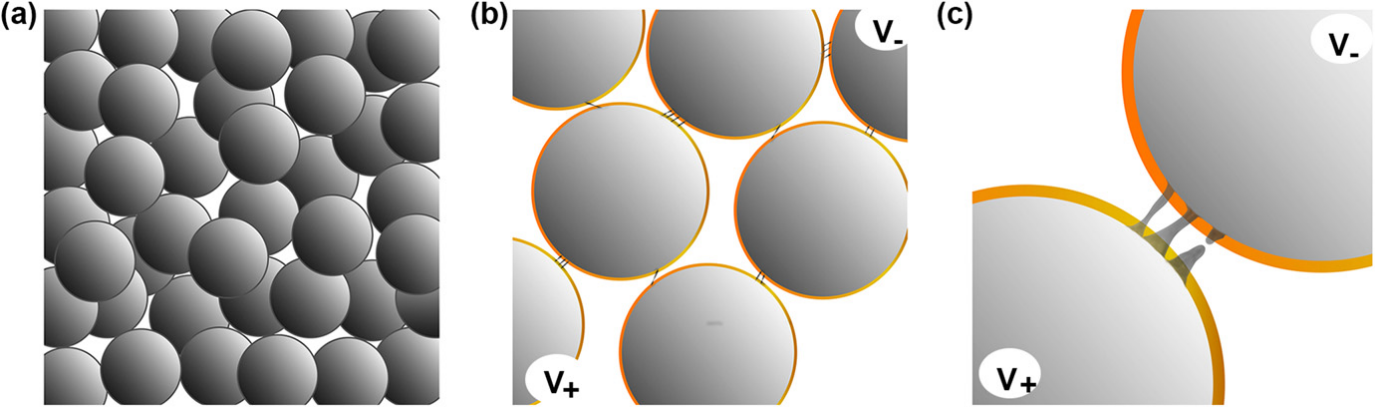
(a) Schematic representation of a silver nanoparticle film. (b) Upon application of an applied bias voltage, the nanoparticles, coated in PVP (shown in orange), are interconnected by sets of conductive filaments which form along the direction of current flow due to electromigration. (c) Several filaments may exist at the junction between two particles and can be broken or thickened as current continues to flow through the film.

The switching mechanism responsible for the observed behavior conceivably gives rise to distinct sets of conductive nanofilament-based pathways (figure 3) that are each associated with a different resistance state [21]. These configurations depend on the applied bias and operating conditions, with the most conductive pathways dominating conduction in the film.

**Figure 4.**
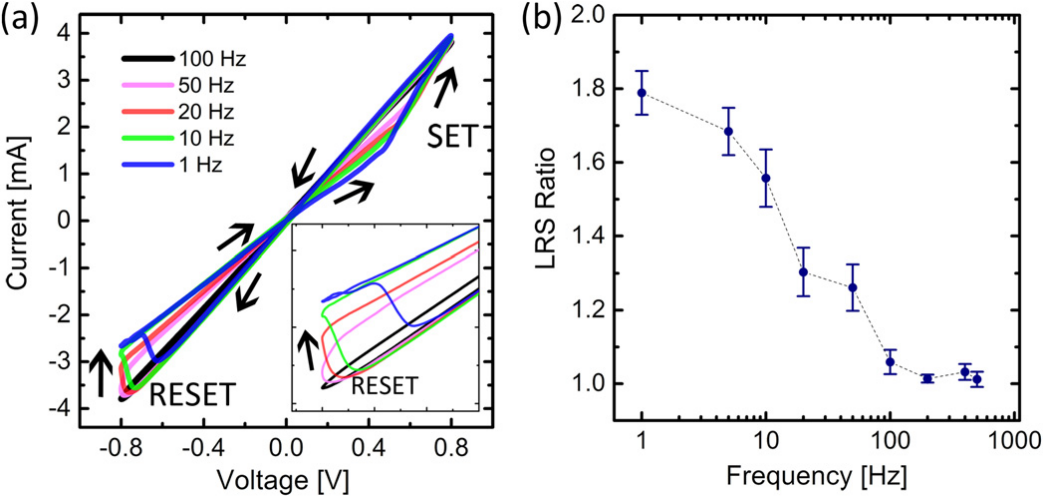
(a) Frequency dependent *I–V* curves with a magnification of the reset process (inset) showed the transition to LRS-2 occurred at a larger voltage with increasing frequency while its resistance steadily decreased. As expected, (b) the LRS ratio decreased to unity at higher frequencies as the second LRS was no longer accessible.

Cycling the applied bias voltage at frequencies less than 100 Hz produced distinct switching between the two LRS (figure 4). Further, frequencies below 50 Hz generally yielded a more rapid increase in resistance as the applied voltage reached its maximum amplitude of 0.8 V. The reset voltage, which we define here as the transition from LRS-1 to LRS-2, increased with frequency until smoother transitions between LRS occurred. As expected from memristive systems the degree of *I–V* hysteresis is significantly lower at higher frequencies [22, 23] (figure 4(a)). Further, the *LRS ratio*, defined here as the ratio of the resistance of LRS-2 to the resistance of LRS-1 and a clear measure of *I–V* hysteresis, tended to unity as the frequency was increased (figure 4(b)). This dependence on sweep frequency is a strong indication that the memristive behavior is due to metallic filament formation and that each set of filamentary substructures which are manifested as distinct resistance states have inherently different structural configurations. The different configurations naturally depend on the operating conditions differently, as suggested by the frequency response of the LRS ratio (figure 4(b)).

**Figure 5.**
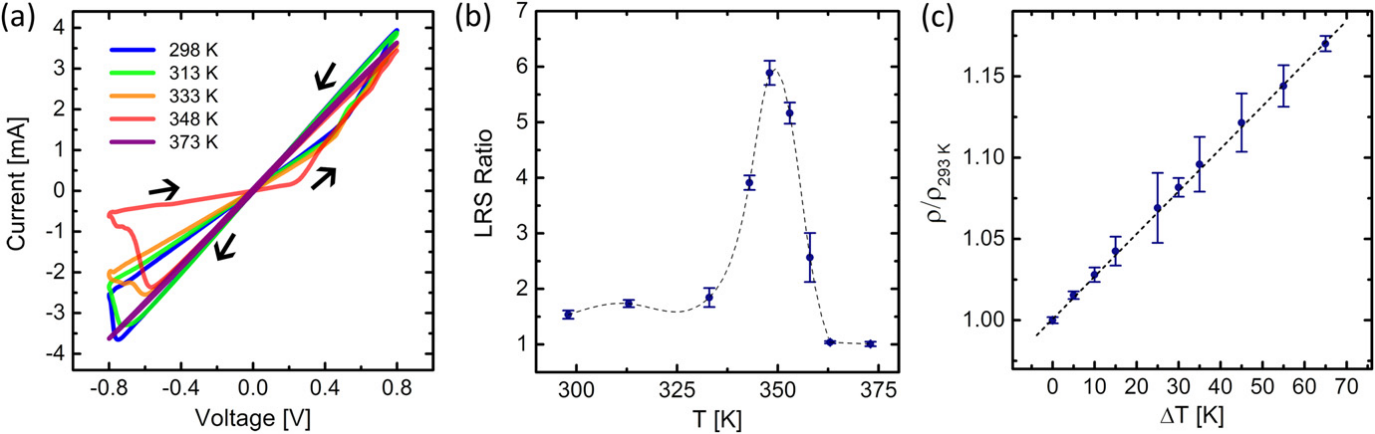
(a) Both LRS resistances increased until the device was heated to the point at which an irreversible effect occurred (between 348 and 373 K). (b) The LRS ratio depended on the operating temperature and increased dramatically at around 350 K (the interpolant is only a guide for the eye). At this point, the second resistance state was no longer observed and (c) the device simply behaved as an ohmic resistor with a change in resistivity proportional to the increase in temperature.

Local temperature variations are another significant factor in electromigration [24] which exacerbates effects such as mass flux between metal particles [15]. A clear dependence of *I–V* hysteresis on temperature was observed (figure 5(a)) and the resistance of both LRS increased when the temperature was raised from 298 to 373 K, though the LRS-2 state was more strongly affected by the change in temperature. This behavior is very strong evidence that conduction in the nanoparticle films is mediated by metallic bridges between particles which result from electromigration effects.

Understanding the conduction to be the effect of metal filaments between nanoparticles, the susceptibility of the second LRS to changes in temperature could be explained by the structural configuration of the LRS-2 state. Being the higher of the two LRS, the LRS-2 would likely comprise more serial connections and fewer parallel connections between silver particles than the LRS-1. The longer effective path length would yield a more significant increase in resistance as it is proportional to the length of the conducting filament for a uniform resistivity and constant nanofilament diameter.

At temperatures above 360 K, the films did not switch between two states but instead exhibited linear *I–V* behavior with a resistance (215 Ω) slightly less than the LRS-1 resistance (225–240 Ω) of the film before the high temperature prohibited access to the LRS-2 state. This suggests that silver filaments through the insulating layer were over-thickened due to increased ionic mobility at higher temperatures, prohibiting switching and short-circuiting the film. After the film was effectively shorted, the change in *I–V* behavior was irreversible and the LRS ratio approached unity (figure 5(b)). The temperature coefficient of resistivity *α* was then calculated (figure 5(c)) using the equation 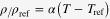 with a 293 K reference temperature and found to be (26 ± 1) × 10^−3^ K^−1^ which is lower than that of bulk silver [25] (38 × 10^−3^ K^−1^). The difference in the value is likely due to the non-uniformity of the material caused by the addition of a polymer coating, or because of the small grain size of the silver nanostructures which has been shown to affect the temperature coefficient of resistivity [26]. From the observed temperature dependence it is concluded that metallic filaments created by electromigration effects are a prominent cause of the observed conduction behavior.

**Figure 6.**
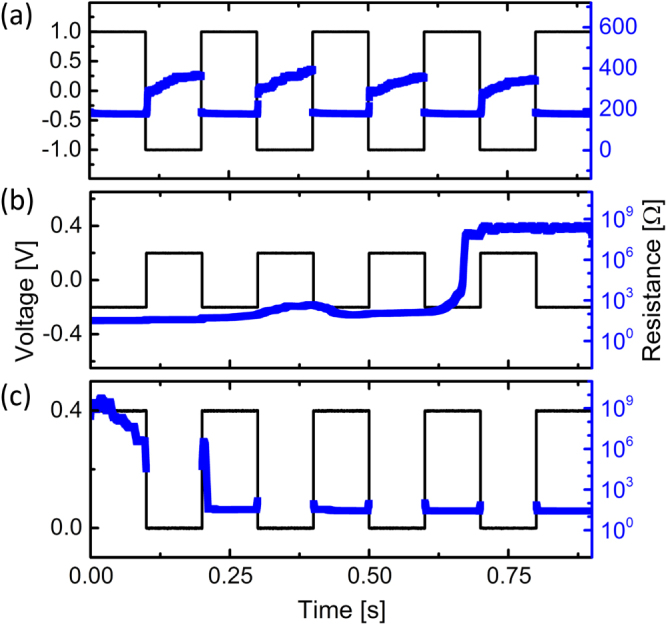
The resistance states of the nanoparticle films were successfully controlled by adjusting the applied square wave bias parameters. (a) The two ON-states were accessed by square wave bias, with LRS-1 being attained during positive bias and LRS-2 accessed when the voltage was negative. (b) The film was turned OFF by applying a symmetric square wave bias with small amplitude. (c) Application of the activating polarity of voltage expectedly resulted in reactivating the film to the LRS-1 state.

Modifying the applied voltage pulse sequence has been shown to be effective at switching between distinct resistance states in other systems [21]. Therefore we controlled the relative amplitudes and duty cycle of the applied square wave voltage to influence the effects of electromigration based filament formation in our nanoparticle films. Each polarity of applied voltage can be associated with a different resistance state: positive—set to LRS-1, negative—reset to LRS-2 (figure 2). To switch between the LRS-1 and LRS-2 states, a 1 V amplitude, 5 Hz, 50% duty cycle bipolar square pulse with zero dc offset was applied as shown in figure 5(a). When the voltage amplitude of the pulse was lowered to 0.2 V, however, the films were successfully switched to the OFF-state (figure 6(b)). Before entering the OFF-state the films were in the LRS-1 state. From the data shown in figure 2(b) we inferred the minimum amplitude required to reactivate the films. The amplitude (0.4 V of the preferred polarity) was determined from the threshold voltage at which the system entered LRS-1. Therefore to reactivate a particular film, a 0.2 V dc offset was included so that the voltage ranged from 0 to 0.4 V which placed it back into the LRS-1 state (figure 6(c)). Because attempts to reactivate the films failed when the peak voltage was below the minimum value (0.4 V), it is likely that formative electromigration effects are necessary for conduction in the nanoparticle films.

## 4. Conclusions

We have fabricated a relatively new type of resistive switching device, composites of silver nanoparticles coated in an insulating polymer TTEG. After a requisite activation step, the films exhibited nonlinear *I–V* behavior that is characteristic of a filament based memristive system and as such could be influenced by the bias sweep frequency. Control over the operational state, either between ON and OFF states with a typical on/off ratio exceeding 1200:1 or between distinct LRS, was readily achieved by manipulating the pulse sequence of applied bias voltage. The accessibility and resistance of these distinct states were adjustable by changing the operating temperature. The dependence on frequency, the polarity dependence of the resistance states and the observed effects of temperature on the conduction behavior of the films are clear indications that filamentary substructures which are formed by electromigration effects are predominantly responsible for the observed conduction behavior. Minimal specifications have been outlined for new memory devices to compete with flash memory and hard disk drives [2] and many have been addressed in this work. Because these nanoparticle films are capable of storing at least two distinguishable resistance states which may be accessed controllably [27], are versatile and easily fabricated, they have great potential for applications in future RRAM technology. Although at room temperature the two available LRS had a resistance ratio between 1 and 2 depending on the sweep frequency (table 1) their stability (figure 2(c)) under normal operating conditions ensured that each state remained completely distinguishable during read-out.

**Table 1.** Dependence of LRS ratio on operational parameters. The temperature measurements were made with a constant sweep frequency of 10 Hz and the frequency measurements were made at a constant temperature of 298 K.

Temperature (K)	LRS ratio	Standard deviation	Frequency (Hz)	LRS ratio	Standard deviation
298	1.54	0.07	1	1.79	0.06
313	1.74	0.07	5	1.68	0.06
333	1.85	0.17	10	1.56	0.08
343	3.91	0.13	20	1.30	0.07
348	5.89	0.22	50	1.26	0.06
353	5.16	0.19	100	1.06	0.03
358	2.57	0.44	200	1.01	0.01
363	1.04	0.02	400	1.03	0.02
373	1.01	0.04	500	1.01	0.02

Future efforts may entail a thorough assessment of endurance to determine whether the nanoparticle films presented here may surpass the 10^6^ cycling limit of flash memory. The minimum write/erase or switching time of the films may also be determined by increasing the temporal resolution of our measurement system and it is expected that rewriting may be achieved within 10^−9^–10^−6^ s.
